# Sleep quality during euthymia in bipolar disorder: the role of clinical features, personality traits, and stressful life events

**DOI:** 10.1186/2194-7511-1-16

**Published:** 2013-09-13

**Authors:** Erika FH Saunders, Danielle M Novick, Julio Fernandez-Mendoza, Masoud Kamali, Kelly A Ryan, Scott A Langenecker, Alan J Gelenberg, Melvin G McInnis

**Affiliations:** Department of Psychiatry, Penn State Milton S. Hershey Medical Center, Penn State College of Medicine, 500 University Drive, P.O. Box 850, Hershey, PA 17033-0850 USA; University of Michigan Department of Psychiatry, Ann Arbor, MI 48109-2700 USA; University of Michigan Depression Center, Ann Arbor, MI 48109-2700 USA; Mood Disorders Clinic, VA Pittsburgh Healthcare System, Pittsburgh, PA 15206 USA; Sleep Research and Treatment Center, Department of Psychiatry, Penn State Milton S. Hershey Medical Center, Penn State College of Medicine, Hershey, PA 17033-0850 USA; University of Illinois at Chicago, Chicago, IL 60612 USA

**Keywords:** Bipolar disorder, Sleep, Neuroticism, Rapid cycling, Stress

## Abstract

**Background:**

Poor sleep quality is known to precede the onset of mood episodes and to be associated with poor treatment outcomes in bipolar disorder (BD). We sought to identify modifiable factors that correlate with poor sleep quality in BD independent of residual mood symptoms.

**Methods:**

A retrospective analysis was conducted to assess the association between the Pittsburgh Sleep Quality Index and clinical variables of interest in euthymic patients with DSM-IV BD (*n* = 119) and healthy controls (HC; *n* = 136) participating in the Prechter Longitudinal Study of Bipolar Disorder. Multivariable linear regression models were constructed to investigate the relationship between sleep quality and demographic and clinical variables in BD and HC participants. A unified model determined independent predictors of sleep quality.

**Results and discussion:**

Euthymic participants with BD and HC differed in all domains. The best fitting unified multivariable model of poor sleep quality in euthymic participants with BD included rapid cycling (*β* = .20, *p* = .03), neuroticism (*β* = .28, *p* = 2 × 10^−3^), and stressful life events (*β* = .20, *p* = .02). Poor sleep quality often persists during euthymia and can be a target for treatment. Clinicians should remain vigilant for treating subjective sleep complaints independent of residual mood symptoms in those sensitive to poor sleep quality, including individuals with high neuroticism, rapid cycling, and recent stressful life events. Modifiable factors associated with sleep quality should be targeted directly with psychosocial or somatic treatment. Sleep quality may be a useful outcome measure in BD treatment studies.

## Background

Bipolar disorder (BD) is a prevalent and chronic psychiatric disorder that is marked by significant impairment in cognitive functioning and impaired quality of life, even during euthymic well intervals (Merikangas et al. 2007; Pattanayak et al. 2012; Ryan et al. 2012; Langenecker et al. 2010; Marshall et al. 2012). For many people with BD, sleep is persistently disrupted, and poor sleep quality is known to precede the onset of both first and recurrent mood episodes (Gruber et al. 2011; Eidelman et al. 2010; Harvey et al. 2005; Jones et al. 2005). In the general population, disturbance of sleep is associated with poor functioning, and insomnia with short sleep duration is associated with poor cognitive functioning and risk for cardiovascular and metabolic diseases (Bixler 2009; Fernandez-Mendoza et al. 2010a; Vgontzas et al. 2009a, b, c). Thus, some morbidity and mortality associated with BD may be related to sleep quality.

Sleep disturbances may be both a cause and a consequence of BD (Talbot et al. 2012; Harvey 2008). Evidence of alterations in the circadian rhythm system comes from genetic and animal models of bipolar disorder (Benedetti et al. 2003, 2007, 2011). Evidence-based somatic treatments (e.g., lithium, melatonin, bright light therapy) and adjunctive psychosocial treatments (e.g., interpersonal social rhythm therapy, behavioral activation) for BD are thought to exert their therapeutic effects, in part, by directly or indirectly shifting, resetting, or stabilizing systems associated with the sleep-wake cycle (Lamont et al. 2007; Frank et al. 2005; McClung 2007).

Little is known about the determinants or correlates of sleep quality during euthymic episodes in BD. In epidemiologic studies of the general population, advancing age, menopausal status, high body mass index (BMI), medical comorbidities, anxiety disorders, stress, and nicotine and substance use are associated with sleep quality (Bixler 2009). In addition, stress and personality factors may play a role in determining sleep quality (Healey et al. 1981; LeBlanc et al. 2007; Marks and Roffwarg 1991; Singareddy et al. 2012; Fernandez-Mendoza et al. 2012a). These factors - aside from advancing age and menopausal status - are generally overrepresented in the BD population (Saunders et al. 2008, 2012; Fagiolini et al. 2002; Swartz and Fagiolini 2012; Prochaska et al. 2011; Dickerson et al. 2013) and thus may be determinants or correlates of sleep quality in the BD population. Against this backdrop, we used data from the baseline visit of the Prechter Longitudinal Study of Bipolar Disorder to understand the effect of clinical features, personality traits, and life events on self-reported sleep quality in a sample of BD subjects who were euthymic and a sample of healthy control (HC) subjects. The Prechter Longitudinal Study is a naturalistic, observational study designed to link detailed clinical assessment of psychiatric and medical history, as well as sleep, stress, environmental factors, substance use, personality, and cognition, to clinical outcomes and genetic and biological data for patients being treated with usual clinical care. We hypothesized that factors in the domains of medical, substance use disorder, and anxiety disorder comorbidities; dimensional/personality traits; life story/trauma; and behavioral domain would show associations with sleep quality in the BD and HC samples similar to those in the general population, and we hypothesized that features of illness in the BD group such as history of suicide attempts, psychosis, mixed episodes, rapid cycling, and high number of episodes would be related to sleep quality.

Our interest in correlates of sleep quality during euthymic episodes in BD is driven by the following questions: Are there modifiable factors that can reliably predict sleep quality and be targeted for treatment, thus reducing the morbidity and mortality associated with poor sleep quality in BD? Can sleep quality be a useful outcome measure in BD treatment studies?

## Methods

### Participants

Patients with BD (i.e., bipolar disorder type I, bipolar disorder type II, schizoaffective disorder, bipolar type or bipolar disorder NOS, Table 1) and HCs with no personal or family history of mood or psychotic disorders were recruited to the Prechter Longitudinal Study of Bipolar Disorder at the University of Michigan, an IRB-approved observational study of outcomes in bipolar disorder (IRBMED HUM000606). Participants in the Prechter Longitudinal Study were seen for a baseline assessment described below and followed long-term with self-reported questionnaires and yearly visits.

**Table 1:** **Clinician-administered and self-report assessment domains**

Domain	Instrument	Time window
Clinical features/comorbidities	DIGS (Nurnberger et al. 1994)	Lifetime
Disease severity	DIGS (Nurnberger et al. 1994)	Lifetime
	HDRS (Hamilton 1960)	Past week
	YMRS (Young et al. 1978)	Past week
Chronotype, sleep, and sleepiness	PSQI (Buysse et al. 1989)	Past month
	ESS (Johns 1991)	None specified
	MCTQ (Roenneberg et al. 2003)	None specified
Dimensional/personality traits	NEO-PI-R (Costa and McCrae 1988)	None specified
Life story/trauma	CTQ (Bernstein et al. 1994)	Lifetime
	LEOS (McKee et al. 2005)	Past six months
	LEC (Johnson and McCutcheon 1980)	Lifetime
	FACES II (Olson et al. 1983)	None specified
	MRCIR (Vinokur and Vanryn 1993)	None specified
Behavioral	AUDIT (Saunders et al. 1993)	Past year
	FTND (Heatherton et al. 1991)	None specified

*DIGS* Diagnostic Interview for Genetic Studies, *HDRS* Hamilton Depression Rating Scale, *YMRS* Young Mania Rating Scale, *PSQI* Pittsburgh Sleep Quality Index, *ESS* Epworth Sleepiness Scale, *MCTQ* Munich Chronotype Questionnaire, *NEO-PI-R* NEO-Personality Index - Revised, *CTQ* Childhood Trauma Questionnaire, *LEOS* Life Events Occurrence Survey, *LEC* Life Events Checklist, *FACES II* Family Adaptability and Cohesion Evaluation Scale II, *MRCIR* Measures Related to Close Interpersonal Relationships Support and Undermining Scale, *AUDIT* Alcohol Use Disorders Identification Test, *FTND* Fagerstrom Test for Nicotine Dependence.

### Analytic cohort

The clinical sample for this investigation included participants recruited at the University of Michigan between 2005 and 2010, and data were extracted in February 2012. The cohort selected for analysis included euthymic patients with BD (*n* = 119) and HCs (*n* = 136) who completed the Pittsburgh Sleep Quality Inventory (PSQI) at baseline. Participants were excluded in the current analysis if they did not have complete data on the PSQI (*n* = 75) or had baseline depression (Hamilton Depression Rating Scale (HDRS) ≥7, *n* = 99) or manic/hypomanic/mixed symptoms (Young Mania Rating Scale (YMRS) ≥7, *n* = 35).

At baseline, clinicians administered the Diagnostic Interview for Genetic Studies (DIGS) (28), HDRS (29), and YMRS (3), and recorded height and weight. The DIGS is a clinician-administered diagnostic interview that includes comprehensive assessment of psychiatric and medical illnesses and includes the ability to assess the lifetime course of mood disorder as well as associated features of mood illness of the individual. Interviewers included physicians, psychologists, and masters-level mental health professionals who completed standardized training in the study instruments, and a best-estimate procedure was used to verify diagnoses (Leckman et al. 1982). Participants completed a baseline set of self-rating questionnaires. Data from the clinical interview and questionnaires were used to assess domains of interest that may relate to sleep quality including sociodemographic factors; clinical features/comorbidities; disease severity; chronotype, sleep, and sleepiness; dimensional/personality traits; life story/trauma history; and behavioral features including substance use (Table 1). Our dependent variable of interest was sleep quality, assessed with the PSQI (Buysse et al. 1989). The PSQI obtains a general measure of sleep quality, and the total score is derived from seven subscales: sleep quality, sleep latency, sleep duration, sleep efficiency, sleep disturbance, sleep medication, and daytime dysfunction. Comparisons of the PSQI subscales were reported between HC and BD groups, and the total PSQI score is used as a dependent variable in analyses. Chronotype was assessed with the Munich Chronotype Questionnaire (MCTQ) (Roenneberg et al. 2003), which assesses preference for sleep and wake times.

### Data and statistical analysis

Values of continuous variables were compared between BD and HC groups using two-sample *t* tests for normally distributed variables or Mann–Whitney *U* tests for variables with a skewed distribution. Categorical variables were compared using Pearson's chi-square test. Linear regression models were constructed for each continuous variable and logistic regression models for each categorical variable to investigate the difference between groups while adjusting for age, sex, and BMI.

Sleep quality (total PSQI score) was correlated with demographic and clinical variables in each of the HC and BD groups first through the use of bivariate comparisons with Pearson's *r* for normally distributed variables or Spearman's *r* for variables that were not normally distributed. To determine independent correlates of poor sleep quality, variables correlated in bivariate comparisons were included in a series of multivariable linear regression models organized by the following domains: sociodemographic factors; clinical features/comorbidities; disease severity; chronotype, sleep, and sleepiness; dimensional/personality traits; life story/trauma history; and behavioral features. Anxiety disorder comorbidity included obsessive-compulsive disorder, panic disorder with or without agoraphobia, social phobia, specific phobia, and agoraphobia without panic. Post-traumatic stress disorder was not included because we did not have a systematic assessment of this disorder. All models at the domain level were adjusted for current depressive symptoms. Final models in each domain were chosen through a backward selection process. Correlates that were independently associated in the domain-wide analyses were selected for a unified multivariable model that was chosen through backward selection.

## Results

### Demographic description of the sample

The average subject with BD (*n* = 119) was a 41-year-old, married (44%), employed (70%) female (66%) with a BMI of 29 (Table 2). The average HC (*n* = 136) was a 32-year-old, not married (74%), employed (84%) female (56%) with a BMI of 25 (Table 2). The age range of the two groups was similar (BD 19 to 65, HC 18 to 66). Approximately one sixth of the HC females had undergone menopause, whereas the rate was one quarter in the BD females. The BD group had significantly more females who were menopausal, had a significantly older group, and a higher BMI. Significantly more BD individuals were married, and significantly more HC were employed or students (Table 2).

**Table 2:** **Sociodemographic characteristics and diagnosis of the sample**

	HC	BD	***p*** value
	(***n*** = 136)	(***n*** = 119)	
Female, *n* (%)	76 (56)	79 (66)	.09
Menopausal, *n* (%)	9 (13)	20 (28)	.02
Age, mean (SD)	32.4 (13.7)	40.5 (12.8)	<1 × 10^−3^
Age, range	18 to 66	19 to 65	
BMI, mean (SD)	25.6 (5.8)	28.9 (6.9)	<1 × 10^−3^
Married, *n* (%)	35 (26)	52 (44)	3 × 10^−3^
Employed/student, *n* (%)	107 (84)	78 (70)	7 × 10^−3^
Bipolar I disorder, *n* (%)		90 (76)	
Bipolar II disorder, *n* (%)		17 (14)	
Bipolar NOS, *n* (%)		10 (8)	
Schizoaffective (BP type), *n* (%)		2 (2)	

### Clinical features of HC and BD subjects

Differences in clinical characteristics between HC and BD subjects are summarized in Table 3, both unadjusted and adjusted for age, sex, and BMI (*p* values reported below are adjusted unless noted otherwise). BD subjects had higher rates of migraine headaches than HC (*p* = 5 × 10^−3^), but not cardiovascular disease (*p* = .08) or diabetes (*p* = .25) after adjusting for age, sex, BMI, and marital and employment/student status. Rates of past history of alcohol use disorders, past history of drug use disorders, and lifetime anxiety disorders (obsessive-compulsive disorder, panic disorder with or without agoraphobia, social phobia, specific phobia, agoraphobia without panic) were 36%, 17%, and 20%, respectively, in the BD group. BD subjects reported a lifetime history of rapid cycling (29%), suicide attempts (33%), and psychosis (59%). A history of mixed episodes, defined by the DSM-IV-TR definition of concurrent manic and depressive episode at the same time, was present in 23% of the sample, and that of mixed symptoms, defined as the presence of subsyndromal manic symptoms during depression or subsyndromal depressive symptoms during mania, was present in 36% of the sample. The average age at onset of BD was 19.2 ± 8.3. The number of episodes was highly skewed to the right, and the BD sample had a median of 5 depressive episodes, 2 manic episodes, and 3 hypomanic episodes. Even in this euthymic sample, current depressive and manic symptoms were higher in the BD than in the HC group (*p* < 1 × 10^−3^).

**Table 3:** **Clinical characteristics of the sample**

Clinical characteristic by domain	HC	BD	***p*** value^a^	***p*** value adjusted^b^
	(***n*** = 136)	(***n*** = 119)		
Medical, substance use disorder, and anxiety disorder comorbidities, *n* (%)				
Cardiovascular disease	8 (6)	24 (21)	1 × 10^−3^	.08
Diabetes	2 (2)	11 (9)	5 × 10^−3^	.25
Migraine	9 (7)	27 (23)	<1 × 10^−3^	5 × 10^−3^
Alcohol use disorders	-	43 (36)	-	-
Drug use disorders	-	20 (17)	-	-
Anxiety disorder	-	24 (20)	-	-
Features of BD, *n* (%)				
Mixed episodes	-	25 (23)	-	-
Rapid cycling	-	34 (29)	-	-
Suicide attempts	-	39 (33)	-	-
Mixed symptoms	-	38 (36)	-	-
Psychosis	-	68 (59)	-	-
Severity/symptom measures, mean (SD)				
Age at onset (years)	-	19.2 (8.3)	-	-
Depression (number of episodes)	-	5 (8)	-	-
Mania (number of episodes)	-	2 (3)	-	-
Hypomania (number of episodes)	-	3 (20)	-	-
Current symptom state				
HDRS 17	0.57 (1.32)	2.06 (1.99)	<1 × 10^−3^	<1 × 10^−3^
HDRS 21 with atypical symptoms	0.76 (1.61)	3.93 (4.39)	<1 × 10^−3^	<1 × 10^−3^
YMRS	0.13 (0.63)	1.21 (1.84)	<1 × 10^−3^	<1 × 10^−3^
Chronotype, sleep, and sleepiness, mean (SD)				
PSQI total score	3.82 (2.7)	6.45 (3.41)	<1 × 10^−3^	<1 × 10^−3^
Sleep quality	0.37 (0.53)	1.03 (1.00)	<1 × 10^−3^	<1 × 10^−3^
Sleep latency	0.74 (0.75)	1.08 (0.98)	3 × 10^−3^	.01
Sleep duration	1.00 (1.09)	0.84 (1.07)	.24	.19
Sleep efficiency	0.30 (0.72)	0.43 (0.79)	.18	.42
Sleep disturbance	0.50 (0.74)	1.13 (1.02)	<1 × 10^−3^	<1 × 10^−3^
Sleep medication	0.70 (0.59)	0.99 (0.73)	<1 × 10^−3^	4 × 10^−3^
Daytime dysfunction	0.21 (0.46)	0.94 (0.91)	<1 × 10^−3^	<1 × 10^−3^
ESS sleepiness	5.38 (3.27)	6.58 (4.18)	.01	.01
MCTQ	3.20 (1.63)	3.03 (1.90)	.46	.40
Dimensional/personality traits, mean (SD)				
NEO-PI neuroticism	44.99 (9.18)	58.38 (13.24)	<1 × 10^−3^	<1 × 10^−3^
NEO-PI extraversion	52.78 (8.63)	50.26 (10.55)	.04	.49
NEO-PI openness	56.59 (10.71)	59.26 (12.07)	.06	7 × 10^−3^
NEO-PI agreeableness	49.75 (10.68)	50.44 (12.51)	.63	.81
NEO-PI conscientiousness	49.20 (10.61)	43.58 (13.27)	<1 × 10^−3^	1 × 10^−3^
Life story/trauma, mean (SD)				
CTQ childhood trauma	34.27 (8.11)	46.60 (17.34)	<1 × 10^−3^	<1 × 10^−3^
LEOS undesirable events	0.85 (1.43)	1.63 (2.17)	1 × 10^−3^	3 × 10^−3^
LEC stressful life events	4.90 (3.77)	4.56 (3.31)	.44	.74
FACES-II cohesion	62.02 (9.39)	55.67 (13.79)	<1 × 10^−3^	<1 × 10^−3^
FACES-II adaptability	46.63 (7.75)	43.54 (10.85)	.01	.03
MRCIR social support	42.70 (6.82)	39.78 (8.90)	<4 × 10^−3^	.12
MRCIR undermining	11.02 (3.64)	13.07 (4.96)	<1 × 10^−3^	2 × 10^−3^
Behavioral, median (IQR)				
Current use				
AUDIT alcohol^c^	0 (4)	3 (5)	.28	
AUDIT drugs^c^	0 (0)	0 (0)	.02	
FTND nicotine dependence^c^	0 (0)	0 (0)	.04	

*DIGS* Diagnostic Interview for Genetic Studies, *HDRS* Hamilton Depression Rating Scale, *YMRS* Young Mania Rating Scale, *PSQI* Pittsburgh Sleep Quality Index, *ESS* Epworth Sleepiness Scale, *MCTQ* Munich Chronotype Questionnaire, *NEO-PI-R* NEO-Personality Index - Revised, *CTQ* Childhood Trauma Questionnaire, *LEOS* Life Events Occurrence Survey, *LEC* Life Events Checklist, *FACES II* Family Adaptability and Cohesion Evaluation Scale II, *MRCIR* Measures Related to Close Interpersonal Relationships Support and Undermining Scale, *AUDIT* Alcohol Use Disorders Identification Test, *FTND* Fagerstrom Test for Nicotine Dependence. ^a^ *p* values from comparisons of continuous variables made using independent *t* tests except for AUDIT alcohol, AUDIT drugs, and FTND nicotine dependence, and *p* values from comparisons of categorical variables made using chi-square tests; ^b^ *p* values from linear regressions including the score of the scale of interest as a dependent variable, with age, sex, BMI, employment/student status, marital status, and euthymic BD/HC group as independent variables; ^c^comparisons between HC and BD are made with Mann–Whitney *U* tests.

BD subjects had poorer sleep quality relative to HC subjects as measured by the PSQI total score as well as the subscales of sleep quality, sleep latency, sleep disturbance, sleep medication, and daytime dysfunction due to sleepiness (*p* < .01). Two of the scales did not differ, including sleep duration (*p* = .19) and sleep efficiency (*p* = .42). Sleepiness was also elevated in the BD group as measured by the Epworth Sleepiness Scale (ESS; *p* = .01). Chronotype did not differ between the BD and HC groups (*p* = .40).

Five-factor personality traits that differed in the BD group included elevated neuroticism and openness and lower conscientiousness (*p* < 7 × 10^−3^). The BD group had higher levels of childhood trauma (*p* < 1 × 10^−3^). The BD group reported higher levels of undesirable events in the last 6 months (*p* = 3 × 10^−3^), but the total number of stressful life events did not differ between BD subjects and HCs (*p* = .74). Family cohesion was lower in the BD group (*p* < 1 × 10^−3^), and family adaptability was lower as well (*p* = .03). Social support was significantly lower in the unadjusted pairwise comparison (unadjusted *p* = 4 × 10^−3^); however, no difference was detected when adjusted for age, sex, BMI, and marital and employment/student status (*p* = .12). Social undermining was higher in the BD group (*p* = 2 × 10^−3^). Current use of alcohol did not differ between groups (unadjusted *p* = .28); however, current use of drugs and nicotine was higher in BD (unadjusted *p* < .04).

### Correlates of sleep quality in HC subjects

Sleep quality was correlated with demographic and clinical variables in the HC group first through the use of Pearson's *r*. Variables that were significantly correlated with poor sleep quality included being married (*r* = .28, *p* = 1 × 10^−3^) and Measures Related to Close Interpersonal Relationships (MRCIR) Undermining Scale (*r* = .25, *p* = 3 × 10^−3^). There was a trend for association with neuroticism (*r* = .16, *p* = .07) and current depressive symptoms (HDRS 21 with atypical, *r* = .17, *p* = .05). Age (*r* = .12, *p* = .18) and female gender (*r* = −.11, *p* = .90) were not associated with sleep quality in HC. To determine independent correlates of sleep quality, variables that were correlated in bivariate comparisons were then included in a series of multivariable linear regression models. The final model was chosen through a backward selection process. MRCIR social undermining was determined to be an independent predictor of sleep quality (*Β* = .20, *p* = .02) when controlled for marital status and current depressive symptoms (*F* = 6.74, *df* = 3, *p* < 1 × 10^−3^; adjusted *R*^2^ = .12).

### Correlates of sleep quality in euthymic BD subjects

To determine independent predictors of sleep quality in a euthymic BD sample, clinical variables of interest were first tested with sleep quality in bivariate correlations. Significant correlates at the bivariate level were included in multivariable regression models by domain, and then a unified multivariable model was created that included all significant correlates from the models by domain (Table 4). In the models by domain, current depressive symptoms were significantly associated with poor sleep quality in each model except personality features. Current depressive symptoms dropped out of the final unified model.

**Table 4:** **Correlates of poor sleep quality in euthymic bipolar subjects (** ***n*** **= 119)**

Domain	Bivariate	Multivariable regression models^a^	Unified multivariable regression model^b^
	***r***	***β*** ( ***p*** )	***β*** ( ***p*** )
Sociodemographic		Adjusted *R* ^2^ = .09	Adjusted *R* ^2^ = .20
Married	−.23 (.01)	−.21 (.02)	-
Age	4 × 10^−3^	-	-
Menopausal status (F)	.01	-	-
Sex	.04	-	-
Employed	−.13	-	-
Medical and anxiety disorder comorbidities		Adjusted *R* ^2^ = .11	
Cardiovascular disease	.20 (.03)	.16 (.08)	-
BMI	.17 (.07)	-	-
Diabetes	.05	-	-
Migraine	.13	-	-
Alcohol use disorders	.16 (.08)	-	-
Drug use disorders	.17 (.07)	.17 (.07)	-
Anxiety disorder	−.09	-	-
Features of BD		Adjusted *R* ^2^ = .15	
Suicide attempts	.31 (1 × 10^−3^)	.22 (.02)	-
Rapid cycling	.30 (1 × 10^−3^)	.20 (.04)	.20 (.03)
Mixed episodes	.20 (.04)	-	-
Mixed symptoms	.13	-	-
Psychosis	.11	-	-
Severity/symptom measures		Adjusted *R* ^2^ = .06	
Age at onset	−.20 (.03)	−.18 (.07)	-
Depression (number of episodes)	.33 (<1 × 10^−3^)	-	-
Mania (number of episodes)	.16 (.09)	-	-
Hypomania (number of episodes)	.23 (.01)	-	-
Current symptom state			
HDRS 17	.11	-	-
HDRS 21 with atypical symptoms	.24 (.01)	-	-
YMRS	−.01	-	-
Chronotype and sleepiness		Adjusted *R* ^2^ = .10	
MCTQ chronotype	.24 (.01)	.23 (.01)	-
ESS sleepiness	.16 (.08)	-	-
Personality assessment		Adjusted *R* ^2^ = .13	
NEO-PI neuroticism	.35 (<1 × 10^−3^)	.31 (1 × 10^−3^)	.28 (2 × 10^−3^)
NEO-PI extraversion	−.01	-	-
NEO-PI openness	.06	-	-
NEO-PI agreeableness	−.01	-	-
NEO-PI conscientiousness	−.17 (.08)	-	-
Life story		Adjusted *R* ^2^ = .11	
LEOS undesirable events	.26 (.01)	.27 (.01)	.20 (.02)
CTQ childhood trauma	.19 (.04)	-	-
LEC stressful life events	.09	-	-
FACES-II cohesion	−.20 (.03)	-	-
FACES-II adaptability	−.15	-	-
MRCIR social support	−.20 (.03)	-	-
MRCIR undermining	.10	-	-
Behavioral			
AUDIT alcohol	−.07	-	-
AUDIT drugs	−.10	-	-
FTND nicotine dependence	.13	-	-

^a^Set of predictors in a series of linear regressions with backward selection in each domain after adjusting for depressive symptoms (*β* ranges from .17 to .26, *p* < .07); ^b^multivariable linear regression, final model obtained through backward selection.

**Figure 1:**
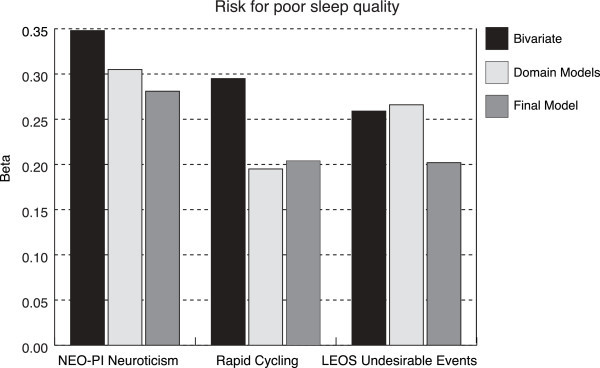
**Independent risk factors for poor sleep quality in euthymic bipolar disorder.**

In the final unified model, independent correlates of sleep quality in the BD group included rapid cycling (*β* = .20, *p* = .03), undesirable events (*β* = .20, *p* = .02), and neuroticism (*β* = .28, *p* = 2 × 10^−3^) (Table 4, Figure 1). Of the features of illness, suicide attempts were the most strongly correlated with sleep quality in the bivariate correlations, and remained significant in the model within the features of illness domain, but dropped out of the unified model. Mixed episodes were also significant in the bivariate correlations, but not in either the features of illness domain model or the unified model. Suicide attempts and rapid cycling were correlated with each other (*r* = .31, *p* = 1 × 10^−3^), and suicide attempts and mixed episodes were correlated with each other (*r* = .32, *p* = 1 × 10^−3^); however, rapid cycling and mixed episodes were not correlated (*r* = .13, *p* = .17). In the life story realm, Life Events Occurrence Survey (LEOS) undesirable events was the strongest correlate in the model by life story domain and remained significant in the unified analysis. Neuroticism was the strongest correlate in the personality assessment domain and in the unified analysis as well.

The only sociodemographic factor that was significant was marital status, but it did not enter into the final model after adjusting for other significant correlates.

Cardiovascular (*p* = .08) and drug use disorders (*p* = .07) were marginally associated with poor sleep quality in the comorbidity domain. In the severity of illness domain, age at onset of BD was significant in the bivariate correlations, and at the domain level, but dropped out of the unified model. Chronotype was also significant at the domain level, but was not present in the unified model.

## Discussion

In this study of euthymic patients with BD, correlates of poor sleep quality independent from residual depressive symptoms were examined in multiple domains that might be expected to affect sleep quality, including sociodemographic factors, medical and anxiety disorder comorbidities, features of BD, severity/symptom measures, chronotype and sleepiness, personality, life story/trauma, and behavioral factors. When domain-wide predictors were included in a final model, independent predictors of poor sleep quality in the BD sample included rapid cycling, high neuroticism, and undesirable events in the past 6 months, and those in the HC sample included social stress.

We can conceptualize poor sleep quality during euthymia in five ways: (1) as a causative trigger for a new mood episode, (2) as a prodromal sign or early symptom of a new mood episode, (3) as a residual symptom of a remitted mood episode, (4) as a distinguishing characteristic of a bipolar phenotype, and (5) as a comorbid sleep disorder/condition. By restricting the analysis to patients in a euthymic period, we designed our approach to study sleep quality as a characteristic of bipolar disorder. Though these subjects were in a traditionally defined ‘euthymic’ period, there was a range of subsyndromal symptoms in this population, consistent with a number of other studies indicating that subsyndromal depressive symptoms are common and predict recurrence (Perlis et al. 2006). The prevalence of subsyndromal symptoms such as poor sleep quality has led some groups to refer to periods without major mood episodes as ‘interepisode’ rather than euthymia (Harvey 2008). In our study, poor sleep quality was correlated with subsyndromal depressive symptoms in euthymic participants when not adjusted for other factors. However, when depressive symptoms were included with other factors affecting sleep quality, the correlation with depressive symptoms was lost, indicating that we found factors that affect sleep quality independently of subsyndromal symptoms. We continue to describe the cohort as euthymic to remain consistent with the bulk of the literature; however, the term interepisode should be considered. Whether poor sleep quality in this population is best categorized as prodromal or residual, treatment targeting the sleep quality outside of targeting depressive symptoms may reduce the substantial impairment and disruption in social and occupational functioning.

We found some consistencies between factors associated with poor sleep quality and psychological and environmental factors that are important predictors of sleep quality in the general population. In the HC group, social undermining predicted poor sleep, whereas in the BD group, association was found between poor sleep and neuroticism and stressful life events. Neuroticism is measured in the widely used five-factor model of personality developed by Costa and McCrae, which also measures extraversion (E), openness (O), agreeableness (A), and conscientiousness (C) (Costa and McCrae 1992). Neuroticism (N) measures the tendency to experience negative affects including fear, sadness, embarrassment, anger, and disgust. Individuals with BD have been shown to have elevated N and O, and low A, C, and E (Barnett et al. 2011). N marks a trait that has been associated with many psychiatric illnesses in addition to BD, including major depressive disorder, anxiety disorders, and personality disorders (Barnett et al. 2011; Costa et al. 2005; Middeldorp et al. 2011; Bagby et al. 2008; Bienvenu et al. 2004). Personality traits have high heritability and may predispose individuals to psychiatric illness; however, there is some indication that personality traits may be affected by psychiatric illness as well (Costa et al. 2005; Christensen and Kessing 2006). We believe that the association between poor sleep quality and neuroticism reported here is a general phenomenon which may be enhanced and exaggerated in BD patients, perhaps due to genetic predisposition, but is not unique to BD or to psychiatrically ill populations.

We found that poor sleep quality was associated with stressful events in euthymic BD and social stress in the HC group, which has also been found in general population samples (Fernandez-Mendoza et al. 2010b; LeBlanc et al. 2007). This is particularly important for BD because social stress has been shown to be greater in BD patients even when euthymic and was shown to be bi-directionally related to disturbed sleep (Eidelman et al. 2012). Affect reactivity in response to stress has also been shown to be greater in euthymic BD than in unipolar depressive disorder or controls (Knowles et al. 2007). Negative mood and sleep have also been shown to have a bi-directional relationship in a euthymic bipolar population; that is, negative mood in the evening may disturb sleep, and disturbed sleep may cause negative mood in the morning (Talbot et al. 2012). Close attention to the role of stress and mood reactivity is warranted in future studies of sleep quality in euthymic BD.

A history of rapid cycling was related to poor sleep quality in the euthymic BD group. Rapid cycling may be associated with sleep quality due to frequent cycling and short euthymic periods between episodes, and similar to stress reactivity, rapid cycling can be conceptualized as both a cause of poor sleep quality and a result of underlying poor sleep quality. In fact, rapid cycling may describe a distinct phenotype with biologically different properties driving both the mood and sleep disturbance. For example, rapid cycling has been linked to panic disorder in familial and genetic studies (MacKinnon and Zamoiski 2006; MacKinnon et al. 2003a, b) and has also been linked to low-activity risk alleles of the catechol-*O*-methyl transferase gene (Papolos et al. 1998; Kirov et al. 1998; Joyce et al. 1995) as well as abnormalities in the hypothalamic-pituitary-thyroid axis (Chakrabarti 2011). In addition, the hypothalamic-pituitary-adrenal (HPA) axis is an important modulator of sleep and the immune system and is abnormally activated in chronic insomnia (Vgontzas and Chrousos 2002; Vgontzas et al. 2004,2007; Basta et al. 2007). One pilot study has shown abnormally high cortisol and high cortisol response to the dexamethasone suppression test in five patients with rapid cycling, regardless of mood state (Watson et al. 2005). Underlying HPA axis dysfunction at baseline or in response to stress may link sleep quality, rapid cycling, and stress reactivity in BD. Rapid cycling is associated with the severity of BD illness and poor outcome (Schneck et al. 2004), and as has been shown in other studies, both rapid cycling and mixed episodes were highly correlated with suicide attempts (Coryell et al. 2003). In this sample, though both rapid cycling and mixed episodes were associated with suicide attempts, rapid cycling and mixed episodes were distinct from each other.

We did not find medical comorbidities, smoking, alcohol or drug use, or anxiety disorders to be significantly associated with sleep quality in HC or BD, when adjusting for other contributing factors. However, we had very few participants who reported active substance use, in part because active substance dependence was an exclusion criterion of the study. In our sample, HCs were younger and had a very low rate of CV disease. Cardiovascular disease and obesity have been associated with insomnia and overall poor sleep quality (Vgontzas et al. 2009a, b, c). In the BD sample, we found a significant association between sleep quality and cardiovascular disease in bivariate correlations and a trend toward association with obesity, but neither was significant in the final model. The domain-wide model identified cardiovascular disease as a stronger predictor of poor sleep quality than obesity.

Diagnosis of anxiety disorder did not affect sleep quality in the BD sample. Though this appears contradictory to the literature showing anxiety affects sleep quality (Fernandez-Mendoza et al. 2012a; Singareddy et al. 2012; Fernandez-Mendoza et al. 2012b), several factors may account for this lack of association. First, anxiety disorder comorbidity was half as common in this sample as other samples that were not restricted to euthymic patients, and in the Prechter cohort, if baseline mood is not restricted to euthymia, rates of anxiety disorders are very similar to published reports (Otto et al. 2006; Saunders et al. 2012). Because anxiety disorders are associated with worse course and severity of illness in BD (Goes et al. 2011; McLean et al. 2011; Saunders et al. 2012), they were underrepresented in our sample, which was restricted to euthymia. Two additional factors include the following: we did not have a systematic assessment of post-traumatic stress disorder (PTSD) included in anxiety disorders on all of our subjects, and we did not have a measurement of general anxiety outside of the specific anxiety disorders noted above. Future study of a measure of general anxiety may elucidate the relationship between anxiety and sleep in BD.

Though we did not have a measure of PTSD for all participants, we did examine the relationship between childhood trauma (CTQ), adult trauma (LEC), and poor sleep. Childhood trauma, but not adult trauma, was higher in the BD group, and associated with sleep quality in bivariate analysis, but recent undesirable events (LEOS) were a stronger predictor of poor sleep quality than childhood trauma in this sample. Recent stresses may have a stronger effect on sleep than childhood traumatic events due to the proximal temporal relationship. An alternate explanation is that individuals with BD who have recurring difficulties from trauma are continuing to have persistent depressive symptoms and may not be represented in this sample.

In the life story/trauma domain, raw scores for social support were lower in the BD group than in the HC group, but this difference was not significant after adjustment for age, sex, BMI, and marital and employment/student status; however, social undermining remained significantly higher in the BD group than in the HC group. This could be due to several reasons - the HC group is much younger, which was likely the reason for fewer marriages in this group. Thus, the BD group may have more individuals who are married, but fewer are employed or students, and may have a restricted social circle, and the marriage may not be a supportive one, but more of the HC are students with a social network.

We expected to find significant differences in sleep quality between women who had undergone menopause and those who have not, as previous population-based studies have shown (Chung and Tang 2006; Blumel et al. 2012; Monterrosa-Castro et al. 2012). One explanation is that the influence of BD on sleep quality may be more important than the effect of menopause; thus, no difference was detected in BD between women pre- and post-menopause. However, in this study, we had only self-reports of menopause, which may mean that peri-menopausal women are included in the pre-menopausal sample.

Limitations of this study include the subjective and retrospective nature of the PSQI. The PSQI measures an aggregate of sleep quality that includes many dimensions that are influenced by many factors, including the use of medications to promote sleep (Buysse et al. 1989). The use of medication may affect the presentation of BD, and thus, it is important to ask how the use of medication may have affected the variables examined in this study. Rapid cycling has been linked to antidepressant use in some studies (Fountoulakis et al. 2013); however, in this sample of euthymic BD subjects, antidepressants were used by a quarter of the participants (*n* = 31) at baseline, and the use was not correlated to the PSQI score (*r* = .03, *p* = .76), neuroticism (*r* = .08, *p* = .40), rapid cycling (*r* = .07, *p* = .46), or stressful events (*r* = .01, *p* = .95). An additional limitation includes the lack of objective physiological measures of physical health other than BMI. The contribution of cardiovascular or physiological measures that may contribute to poor sleep quality is likely underestimated in this study. In addition, our control group was younger than the BD group. However, we adjusted for age when comparing the HC and BD groups. The study strengths include a large sample of patients with well-characterized BD, as well as dimensional measures of sleep, personality, psychosocial stress, and behavioral measures.

## Conclusions

Poor sleep quality is associated with depressive and manic episodes but often persists between episodes. Treatment of poor sleep quality between episodes is a frequent clinical problem, and targets for improving sleep quality, in addition to targeting residual mood symptoms, may improve outcomes in BD. Here, we present correlates of poor sleep quality that may be used to target treatments. Clinicians should remain vigilant for treating subjective sleep complaints independent of mood complaints in BD patients sensitive to poor sleep quality, including individuals with high neuroticism and rapid cycling. Reactivity to stressful life events during euthymia can be a target for psychotherapy, and modification may improve stress response. Association between poor sleep quality and rapid cycling emphasizes the importance of treatment of frequently cycling moods and improving sleep quality as mood and sleep quality each impact each other. BD patients with recent life stressors, history of rapid cycling, and high neuroticism are at elevated risk of experiencing poor sleep quality. Areas for further study include identifying correlates of specific subjective sleep complaints and using a home-based objective sleep measure such as actigraphy to describe sleep-related risk factors for poor outcome in BD (Faurholt-Jepsen et al. 2012; Kaplan et al. 2012; Jones et al. 2005).

## References

[CR1] Bagby RM, Quilty LC, Segal ZV, McBride CC, Kennedy SH, Costa PT (2008). Personality and differential treatment response in major depression: a randomized controlled trial comparing cognitive-behavioural therapy and pharmacotherapy. Can J Psychiatry.

[CR2] Barnett JH, Huang J, Perlis RH, Young MM, Rosenbaum JF, Nierenberg AA (2011). Personality and bipolar disorder: dissecting state and trait associations between mood and personality. Psychol Med.

[CR3] Basta M, Chrousos GP, Vela-Bueno A, Vgontzas AN (2007). Chronic insomnia and stress system. Sleep Medicine Clinics.

[CR4] Benedetti F, Serretti A, Colombo C, Barbini B, Lorenzi C, Campori E (2003). Influence of CLOCK gene polymorphism on circadian mood fluctuation and illness recurrence in bipolar depression. Am J Med Genet B.

[CR5] Benedetti F, Dallaspezia S, Fulgosi MC, Lorenzi C, Serretti A, Barbini B (2007). Actimetric evidence that CLOCK 3111 T/C SNP influences sleep and activity patterns in patients affected by bipolar depression. Am J Med Genet B.

[CR6] Bernstein DP, Fink L, Handelsman L, Foote J, Lovejoy M, Wenzel K (1994). Initial reliability and validity of a new retrospective measure of child abuse and neglect. Am J Psychiatry.

[CR7] Bienvenu OJ, Samuels JF, Costa PT, Reti IM, Eaton WW, Nestadt G (2004). Anxiety and depressive disorders and the five-factor model of personality: a higher- and lower-order personality trait investigation in a community sample. Depress Anxiety.

[CR8] Bixler E (2009). Sleep and society: an epidemiological perspective. Sleep Med.

[CR9] Blumel JE, Cano A, Mezones-Holguin E, Baron G, Bencosme A, Benitez Z (2012). A multinational study of sleep disorders during female mid-life. Maturitas.

[CR10] Buysse DJ, Reynolds CF, Monk TH, Berman SR, Kupfer DJ (1989). The Pittsburgh Sleep Quality Index: a new instrument for psychiatric practice and research. Psychiatry Res.

[CR11] Chakrabarti S (2011). Thyroid functions and bipolar affective disorder. J Thyroid Res.

[CR12] Christensen MV, Kessing LV (2006). Do personality traits predict first onset in depressive and bipolar disorder?. Nord J Psychiatry.

[CR13] Chung KF, Tang MK (2006). Subjective sleep disturbance and its correlates in middle-aged Hong Kong Chinese women. Maturitas.

[CR14] Coryell W, Solomon D, Turvey C, Keller M, Leon AC, Endicott J (2003). The long-term course of rapid-cycling bipolar disorder. Arch Gen Psychiatry.

[CR15] Costa PT, McCrae RR (1988). Personality in adulthood: a six-year longitudinal study of self-reports and spouse ratings on the NEO Personality Inventory. J Pers Soc Psychol.

[CR16] Costa PT, McCrae RR (1992). Revised NEO Personality Inventory (NEO PI-R) and NEO Five-Factor Inventory (NEO-FFI) Professional Manual.

[CR17] Costa PT, Bagby RM, Herbst JH, McCrae RR (2005). Personality self-reports are concurrently reliable and valid during acute depressive episodes. J Affect Disord.

[CR18] Dickerson F, Stallings CR, Origoni AE, Vaughan C, Khushalani S, Schroeder J (2013). Cigarette smoking among persons with schizophrenia or bipolar disorder in routine clinical settings, 1999–2011. Psychiatr Serv.

[CR19] Eidelman P, Talbot LS, Gruber J, Harvey AG (2010). Sleep, illness course, and concurrent symptoms in inter-episode bipolar disorder. J Behav Ther Exp Psychiatry.

[CR20] Eidelman P, Gershon A, Kaplan K, McGlinchey E, Harvey AG (2012). Social support and social strain in inter-episode bipolar disorder. Bipolar Disord.

[CR21] Fagiolini A, Frank E, Houck PR, Mallinger AG, Swartz HA, Buysse DJ (2002). Prevalence of obesity and weight change during treatment in patients with bipolar I disorder. J Clin Psychiatry.

[CR22] Faurholt-Jepsen M, Brage S, Vinberg M, Christensen EM, Knorr U, Jensen HM (2012). Differences in psychomotor activity in patients suffering from unipolar and bipolar affective disorder in the remitted or mild/moderate depressive state. J Affect Disord.

[CR23] Fernandez-Mendoza J, Calhoun S, Bixler EO, Pejovic S, Karataraki M, Liao D (2010). Insomnia with objective short sleep duration is associated with deficits in neuropsychological performance: a general population study. Sleep.

[CR24] Fernandez-Mendoza J, Vela-Bueno A, Vgontzas AN, Ramos-Platon MJ, Olavarrieta-Bernardino S, Bixler EO (2010). Cognitive-emotional hyperarousal as a premorbid characteristic of individuals vulnerable to insomnia. Psychosom Med.

[CR25] Fernandez-Mendoza J, Vgontzas AN, Bixler EO, Singareddy R, Shaffer ML, Calhoun SL (2012). Clinical and polysomnographic predictors of the natural history of poor sleep in the general population. Sleep.

[CR26] Fernandez-Mendoza J, Vgontzas AN, Liao D, Shaffer ML, Vela-Bueno A, Basta M (2012). Insomnia with objective short sleep duration and incident hypertension: the Penn State Cohort. Hypertension.

[CR27] Fountoulakis KN, Kontis D, Gonda X, Yatham LN (2013). A systematic review of the evidence on the treatment of rapid cycling bipolar disorder. Bipolar Disord.

[CR28] Frank E, Kupfer DJ, Thase ME, Mallinger AG, Swartz HA, Fagiolini AM (2005). Two-year outcomes for interpersonal and social rhythm therapy in individuals with bipolar I disorder. Arch Gen Psychiatry.

[CR29] Goes FS, McCusker MM, Bienvenu OJ, Mackinnon DF, Mondimore FM, Schweizer B (2011). Co-morbid anxiety disorders in bipolar disorder and major depression: familial aggregation and clinical characteristics of co-morbid panic disorder, social phobia, specific phobia and obsessive-compulsive disorder. Psychol Med.

[CR30] Gruber J, Miklowitz DJ, Harvey AG, Frank E, Kupfer D, Thase ME (2011). Sleep matters: sleep functioning and course of illness in bipolar disorder. J Affect Disord.

[CR31] Hamilton M (1960). A rating scale for depression. J Neurol Neurosurg Psychiatry.

[CR32] Harvey AG (2008). Sleep and circadian rhythms in bipolar disorder: seeking synchrony, harmony, and regulation. Am J Psychiatry.

[CR33] Harvey AG, Schmidt DA, Scarna A, Semler CN, Goodwin GM (2005). Sleep-related functioning in euthymic patients with bipolar disorder, patients with insomnia, and subjects without sleep problems. Am J Psychiatry.

[CR34] Healey ES, Kales A, Monroe LJ, Bixler EO, Chamberlin K, Soldatos CR (1981). Onset of insomnia: role of life-stress events. Psychosom Med.

[CR35] Heatherton TF, Kozlowski LT, Frecker RC, Fagerstrom KO (1991). The Fagerstrom Test for Nicotine Dependence: a revision of the Fagerstrom Tolerance Questionnaire. Br J Addict.

[CR36] Johns MW (1991). A new method for measuring daytime sleepiness: the Epworth sleepiness scale. Sleep.

[CR37] Johnson JH, McCutcheon S, Sarason IE, Speilberger CD (1980). Assessing life stress in older children and adolescents: preliminary findings with the life events checklist. Stress and Anxiety.

[CR38] Jones SH, Hare DJ, Evershed K (2005). Actigraphic assessment of circadian activity and sleep patterns in bipolar disorder. Bipolar Disord.

[CR39] Joyce PR, Fergusson DM, Woollard G, Abbott RM, Horwood LJ, Upton J (1995). Urinary catecholamines and plasma hormones predict mood state in rapid cycling bipolar affective disorder. J Affect Disord.

[CR40] Kaplan KA, Talbot LS, Gruber J, Harvey AG (2012). Evaluating sleep in bipolar disorder: comparison between actigraphy, polysomnography, and sleep diary. Bipolar Disord.

[CR41] Kirov G, Murphy KC, Arranz MJ, Jones I, McCandles F, Kunugi H (1998). Low activity allele of catechol-O-methyltransferase gene associated with rapid cycling bipolar disorder. Mol Psychiatry.

[CR42] Knowles R, Tai S, Jones SH, Highfield J, Morriss R, Bentall RP (2007). Stability of self-esteem in bipolar disorder: comparisons among remitted bipolar patients, remitted unipolar patients and healthy controls. Bipolar Disord.

[CR43] Lamont EW, Legault-Coutu D, Cermakian N, Boivin DB (2007). The role of circadian clock genes in mental disorders. Dialogues Clin Neurosci.

[CR44] Langenecker SA, Saunders EF, Kade AM, Ransom MT, McInnis MG (2010). Intermediate: cognitive phenotypes in bipolar disorder. J Affect Disord.

[CR45] LeBlanc M, Beaulieu-Bonneau S, Merette C, Savard J, Ivers H, Morin CM (2007). Psychological and health-related quality of life factors associated with insomnia in a population-based sample. J Psychosom Res.

[CR46] Leckman JF, Sholomskas D, Thompson WD, Belanger A, Weissman MM (1982). Best estimate of lifetime psychiatric diagnosis: a methodological study. Arch Gen Psychiatry.

[CR47] MacKinnon DF, Zamoiski R (2006). Panic comorbidity with bipolar disorder: what is the manic-panic connection?. Bipolar Disord.

[CR48] MacKinnon DF, Zandi PP, Gershon E, Nurnberger JI, Reich T, DePaulo JR (2003). Rapid switching of mood in families with multiple cases of bipolar disorder. Arch Gen Psychiatry.

[CR49] MacKinnon DF, Zandi PP, Gershon ES, Nurnberger JI, DePaulo JR (2003). Association of rapid mood switching with panic disorder and familial panic risk in familial bipolar disorder. Am J Psychiatry.

[CR50] Marks GA, Roffwarg HP (1991). Cholinergic modulation of responses to glutamate in the thalamic reticular nucleus of the anesthetized rat. Brain Res.

[CR51] Marshall DF, Walker SJ, Ryan KA, Kamali M, Saunders EF, Weldon AL (2012). Greater executive and visual memory dysfunction in comorbid bipolar disorder and substance use disorder. Psychiatry Res.

[CR52] McClung CA (2007). Clock genes and bipolar disorder: implications for therapy. Pharmacogenomics.

[CR53] McClung CA (2011). Circadian rhythms and mood regulation: insights from pre-clinical models. Eur Neuropsychopharmacol.

[CR54] McKee SAAM, Kochetkova A, Maciejewski P, O'Malley S, Krishnan-Sarin S, Mazure CM (2005). A new measure for assessing the impact of stressful events on smoking behavior. Prague. Annual Meeting of the Society for Research on Nicotine and Tobacco; March 20–23 2005: Prague, Czech Republic.

[CR55] McLean CP, Asnaani A, Litz BT, Hofmann SG (2011). Gender differences in anxiety disorders: prevalence, course of illness, comorbidity and burden of illness. J Psychiatr Res.

[CR56] Merikangas KR, Akiskal HS, Angst J, Greenberg PE, Hirschfeld RM, Petukhova M (2007). Lifetime and 12-month prevalence of bipolar spectrum disorder in the National Comorbidity Survey replication. Arch Gen Psychiatry.

[CR57] Middeldorp CM, de Moor MH, McGrath LM, Gordon SD, Blackwood DH, Costa PT (2011). The genetic association between personality and major depression or bipolar disorder. A polygenic score analysis using genome-wide association data. Transl Psychiatry.

[CR58] Monterrosa-Castro A, Marrugo-Florez M, Romero-Perez I, Fernandez-Alonso AM, Chedraui P, Perez-Lopez FR (2012). Assessment of sleep quality and correlates in a large cohort of Colombian women around menopause. Menopause.

[CR59] Nurnberger JI, Blehar MC, Kaufmann CA, York-Cooler C, Simpson SG, Harkavy-Friedman J (1994). Diagnostic interview for genetic studies. Rationale, unique features, and training. NIMH Genetics Initiative. Arch Gen Psychiatry.

[CR60] Olson DH, Russell CS, Sprenkle DH (1983). Circumplex model of marital and family systems: VI. Theoretical update. Fam Process.

[CR61] Otto MW, Simon NM, Wisniewski SR, Miklowitz DJ, Kogan JN, Reilly-Harrington NA, Frank E, Nierenberg AA, Marangell LB, Sagduyu K, Weiss RD, Miyahara S, Thase ME, Sachs GS, Pollack MH, STEP-BD Investigators (2006). Prospective 12-month course of bipolar disorder in out-patients with and without comorbid anxiety disorders. Brit J Psychiat: J Ment Sci.

[CR62] Papolos DF, Veit S, Faedda GL, Saito T, Lachman HM (1998). Ultra-ultra rapid cycling bipolar disorder is associated with the low activity catecholamine-O-methyltransferase allele. Mol Psychiatry.

[CR63] Pattanayak RD, Sagar R, Mehta M (2012). Neuropsychological performance in euthymic Indian patients with bipolar disorder type I: correlation between quality of life and global functioning. Psychiatry Clin Neurosci.

[CR64] Perlis RH, Ostacher MJ, Patel JK, Marangell LB, Zhang H, Wisniewski SR (2006). Predictors of recurrence in bipolar disorder: primary outcomes from the Systematic Treatment Enhancement Program for Bipolar Disorder (STEP-BD). Am J Psychiatry.

[CR65] Prochaska JJ, Reyes RS, Schroeder SA, Daniels AS, Doederlein A, Bergeson B (2011). An online survey of tobacco use, intentions to quit, and cessation strategies among people living with bipolar disorder. Bipolar Disord.

[CR66] Roenneberg T, Wirz-Justice A, Merrow M (2003). Life between clocks: daily temporal patterns of human chronotypes. J Biol Rhythms.

[CR67] Ryan KA, Vederman AC, McFadden EM, Weldon AL, Kamali M, Langenecker SA (2012). Differential executive functioning performance by phase of bipolar disorder. Bipolar Disord.

[CR68] Saunders JB, Aasland OG, Babor TF, de la Fuente JR, Grant M (1993). Development of the Alcohol Use Disorders Identification Test (AUDIT): WHO collaborative project on early detection of persons with harmful alcohol consumption–II. Addiction.

[CR69] Saunders EH, Scott LJ, McInnis MG, Burmeister M (2008). Familiality and diagnostic patterns of subphenotypes in the National Institutes of Mental Health bipolar sample. Am J Med Genet B.

[CR70] Saunders EF, Fitzgerald KD, Zhang P, McInnis MG (2012). Clinical features of bipolar disorder comorbid with anxiety disorders differ between men and women. Depress Anxiety.

[CR71] Schneck CD, Miklowitz DJ, Calabrese JR, Allen MH, Thomas MR, Wisniewski SR (2004). Phenomenology of rapid-cycling bipolar disorder: data from the first 500 participants in the systematic treatment enhancement program. Am J Psychiatry.

[CR72] Singareddy R, Vgontzas AN, Fernandez-Mendoza J, Liao D, Calhoun S, Shaffer ML (2012). Risk factors for incident chronic insomnia: a general population prospective study. Sleep Med.

[CR73] Swartz HA, Fagiolini A (2012). Cardiovascular disease and bipolar disorder: risk and clinical implications. J Clin Psychiatry.

[CR74] Talbot LS, Stone S, Gruber J, Hairston IS, Eidelman P, Harvey AG (2012). A test of the bidirectional association between sleep and mood in bipolar disorder and insomnia. J Abnorm Psychol.

[CR75] Vgontzas AN, Chrousos GP (2002). Sleep, the hypothalamic-pituitary-adrenal axis, and cytokines: multiple interactions and disturbances in sleep disorders. Endocrinol Metab Clin North Am.

[CR76] Vgontzas AN, Zoumakis E, Bixler EO, Lin HM, Follett H, Kales A (2004). Adverse effects of modest sleep restriction on sleepiness, performance, and inflammatory cytokines. J Clin Endocrinol Metab.

[CR77] Vgontzas AN, Pejovic S, Zoumakis E, Lin HM, Bentley CM, Bixler EO (2007). Hypothalamic-pituitary-adrenal axis activity in obese men with and without sleep apnea: effects of continuous positive airway pressure therapy. J Clin Endocrinol Metab.

[CR78] Vgontzas AN, Liao D, Bixler EO (2009). Insomnia and hypertension. Sleep.

[CR79] Vgontzas AN, Liao D, Bixler EO, Chrousos GP, Vela-Bueno A (2009). Insomnia with objective short sleep duration is associated with a high risk for hypertension. Sleep.

[CR80] Vgontzas AN, Liao D, Pejovic S, Calhoun S, Karataraki M, Bixler EO (2009). Insomnia with objective short sleep duration is associated with type 2 diabetes: a population-based study. Diabetes Care.

[CR81] Vinokur AD, Vanryn M (1993). Social support and undermining in close relationships: their independent effects on the mental health of unemployed persons. J Pers Soc Psychol.

[CR82] Watson S, Thompson JM, Malik N, Ferrier IN, Young AH (2005). Temporal stability of the dex/CRH test in patients with rapid-cycling bipolar I disorder: a pilot study. Aust N Z J Psychiatry.

[CR83] Young RC, Biggs JT, Ziegler VE, Meyer DA (1978). A rating scale for mania: reliability, validity and sensitivity. Br J Psychiatry.

